# Distinct immune memory induced by SARS-CoV-2 in convalescent liver transplant recipients

**DOI:** 10.3389/fimmu.2025.1420150

**Published:** 2025-04-02

**Authors:** Mengcheng Liu, Feng Wu, Binwei Duan, Yuxuan Zhang, Wenjing Wang, Zhuangzhuang Chen, Yibo Sun, Gongming Zhang, Yifei Wang, Yueyi Sun, Yabo Ouyang, Guangming Li

**Affiliations:** ^1^ Department of General Surgery Center, Beijing YouAn Hospital, Capital Medical University, Beijing Institute of Hepatology, Beijing, China; ^2^ Clinical Center for Liver Cancer, Capital Medical University, Beijing, China; ^3^ Beijing Engineering Research Center for Precision Medicine and Transformation of Hepatitis and Liver Cancer, Beijing, China

**Keywords:** liver transplant recipients, SARS-CoV-2 convalescent, neutralizing antibodies, immune memory, T cell phenotype

## Abstract

The understanding of how the host immune response differs in T-cell phenotype and memory formation during SARS-CoV-2 infection in liver transplant recipients (LTRs) remains limited. LTRs who recovered from COVID-19 infection without prior vaccination represent a unique population for studying immune responses to SARS-CoV-2. Six LTRs with positive neutralizing antibodies (nAb+) and six LTRs with negative nAb (nAb-) were included at 6 months following COVID-19 infection. It was found that nAb+ LTRs had higher anti-RBD IgG titers and greater neutralizing percent inhibition compared to nAb- LTRs. Fifteen T-cell subsets were identified in COVID-19 convalescent LTRs, and it was shown that only terminal effector CD8+ - 3 decreased in the nAb+ group, while elevated IL-10 expression levels were found in the nAb- group. After stimulation with the SARS-CoV-2 XBB spike peptide pool *in vitro*, it was observed that the nAb+ group exhibited an increase in effector memory CD4+ cells with lower PD-1 expression, a reduction in effector memory CD4+ - 2 cells, and terminal effector CD8+ - 3 cells, while the nAb- group showed high expression of CTLA-4 and IL-10 in terminal effector CD8+ - 3 cells. Four SARS-CoV-2-specific T-cell subsets were identified, with high expression of TNF-α and IFN-γ in terminal effector CD8+ - 1 and terminal effector CD8+ - 2 cells in both groups. Perforin was mainly detected in terminal effector CD8+ - 2 cells in nAb+ LTRs. In addition to these proportional differences, stem cell memory CD4+ cells with higher IL-17A expression and stem cell memory CD8+ cells with higher CTLA-4 expression were also found in nAb- LTRs. These findings suggest that LTRs who developed nAb+ following SARS-CoV-2 infection exhibit stronger T-cell responses, with more robust immune activation and memory recall, compared to nAb- LTRs. This study underscores the importance of understanding T-cell responses during SARS-CoV-2 recovery for guiding vaccination strategies and managing immunity in LTRs.

## Introduction

Immunosuppression and comorbidities make liver transplant recipients (LTRs) a vulnerable population with a markedly elevated risk of symptomatic SARS-CoV-2 infection compared to the general population (1). To date, most studies in LTRs have evaluated cellular and humoral immunity against SARS-CoV-2 following two doses of mRNA vaccines (2, 3). LTRs exhibit a lower immune response to SARS-CoV-2 vaccination, as evidenced by a lower rate of seroconversion and lower antibody titers compared to immunocompetent patients or healthy donors (4–6). Changes in the humoral immune response after SARS-CoV-2 infection in LTRs have also been assessed, showing that anti-nucleocapsid IgG antibodies have lower durability and faster decay within the first 6 months after infection in LTRs, compared with immunocompetent patients (7). In addition to neutralizing antibodies (nAb), optimal immunity to SARS-CoV-2 requires strong T-cell responses to protect against both current SARS-CoV-2 strains and emerging variants (8).

Several studies have investigated immune responses in LTRs vaccinated with SARS-CoV-2 inactivated vaccines, but the immune mechanisms involved in their response to natural infection remain poorly understood (6, 9, 10). In particular, T-cell mediated immunity, especially T memory cell formation, is poorly understood in LTRs who have recovered from natural COVID-19 infection without prior vaccination. This gap in knowledge is significant, as T cells play a crucial role in long-term immunity and protection against reinfection, particularly for immunocompromised individuals such as LTRs (11). Despite advances in vaccination strategies, understanding the differences in T-cell responses between nAb+ and nAb- LTRs remains an important, unanswered question. It is essential to understand how these T-cell subsets respond to SARS-CoV-2 infection and how this impacts vaccine efficacy, reinfection risk, and overall immune health in LTRs.

Our study aims to address these knowledge gaps by comparing T-cell responses in LTRs who experienced their first COVID-19 infection without prior vaccination. Specifically, we investigate differences in T-cell phenotypes and memory formation based on post-infection nAb status, providing valuable insights into how immune memory is shaped in this unique patient population. To achieve this, we used PBMCs derived from recovered COVID-19 LTRs to assess memory responses by evaluating antibody production, T-cell phenotyping, and intracellular cytokine levels. Understanding these immune mechanisms is critical for guiding clinical strategies, particularly for LTRs who may be unwilling to receive the COVID-19 vaccine or may experience diminished vaccine responses due to their immunosuppressive therapy. By addressing these unknowns, our study aims to improve the clinical management of LTRs in the context of COVID-19, offering new perspectives on immune monitoring and potential therapeutic approaches for this vulnerable group.

## Materials and methods

### Study participants

Six LTRs with nAb+ and six LTRs with nAb-, matched according to clinical variables associated with LTRs, were included 6 months following coronavirus disease 2019 (COVID-19) infection without prior SARS-CoV-2 vaccination. A comparison of the clinical data for the LTRs is presented in **Table 1**. No significant differences were observed between the two groups of LTRs in terms of age, gender, time from transplantation to the first SARS-CoV-2 infection, types of immunosuppressants, or comorbidities (**Table 1**, all P > 0.05). Plasma was obtained by centrifuging blood samples at 3500 rpm for 5 minutes and frozen at –80°C for further analysis. PBMCs were isolated by density gradient centrifugation using lymphocyte separation medium (Corning). After isolation, the cells were cryopreserved in fetal bovine serum (Corning) with 10% dimethyl sulfoxide (DMSO; Sigma-Aldrich) until use. The viability of cryopreserved PBMCs was assessed using the Trypan blue exclusion method, and viability was consistently above 85% prior to analysis. All clinical datas of LTRs within 4 weeks before sample collection were retrospectively reviewed. The studies involving human participants were approved by the Ethics Committee of Beijing YouAn Hospital ([2021]083). The studies were conducted in accordance with local legislation and institutional requirements. All participants provided written informed consent to participate in this study. No potentially identifiable images or data are presented in this study.

**Table 1:** Comparison of clinical characteristics in the liver transplantation recipients.

	nAb+ (n = 6)	nAb- (n = 6)	*P*-Value
Age (years)	64.9 (55.3-69.0)	51.0 (46.2-63.2)	0.057^a^
Gender (Male)	6	6	1.00^b^
Duration from LT to first SARS-CoV-2 infection (years)	8.5 (1.6-15.4)	4.3 (4.1-11.4)	1.000^a^
Tacrolimus daily dose (mg/day)	1.0 (1.0-3.0)	1.0 (0-2.0)	0.315^a^
MMF/MPA or not	2:4	1:5	1.000^b^
Drug concentration of sirolimus	5.1 (3.9-5.1)	4.3 (0.9-4.9)	0.827^a^
Drug concentration of tacrolimus	3.3 (1.8-4.8)	1.9 (1.0-7.0)	0.784^a^
Diabetes or not	3:3	0:6	0.182^b^

LT, liver transplant; MMF, mycophenolate mofetil; MPA, mycophenolic acid. a. Kruskal–Wallis test, b. Fisher’s exact test.

### Anti-SARS-CoV-2 neutralizing antibody detection

Anti-SARS-CoV-2 neutralizing antibody levels were determined using the Anti-SARS-CoV-2 Neutralizing Antibody Titer Serologic Assay Kit (ACROBiosystems, USA) as previously described (6). The cutoff value was set at 20% signal inhibition. A neutralizing percent inhibition (NPI) ≥20% indicated the presence of Anti-SARS-CoV-2 neutralizing antibodies (nAb+), while an NPI <20% indicated the absence of neutralizing antibodies (nAb-).

### Detection of anti-SARS-CoV-2 antibody IgG titer

The IgG titers of antibodies against the structural protein RBD were determined using the indirect ELISA kit (ACROBiosystems, USA) according to the manufacturer’s instructions. Each value obtained was the average of three independent biological replicates.

### T cells stimulation

1 × 10^6^ cryopreserved PBMCs were cultured in a 6-well plate (Corning) with TexMACS™ GMP Medium, containing 100 U/mL penicillin and 100 μg/mL streptomycin. The cells were then stimulated with 1 μg/mL SARS-CoV-2 Spike Omicron XBB peptide pool (QYAOBIO, catalog number 4890000013, China) at 37°C and 5% CO_2_ for 24 hours, following the manufacturer’s guidelines for optimal T-cell stimulation. A cell activation cocktail kit, consisting of PMA (phorbol 12-myristate 13-acetate) and ionomycin (BioLegend, catalog number 423301, USA), was used as the positive control, following the manufacturer’s instructions, in a separate group. DMSO (dimethyl sulfoxide) at a final concentration of 0.02% was used as the negative control in another group. Brefeldin A (BioLegend) was added for an additional 4 hours to facilitate intracellular cytokine detection by mass cytometry.

### Mass cytometry and data analysis

PBMCs from LTRs were incubated with 1 mM cisplatin (198-Pt, Fluidigm, USA) for 2 minutes to evaluate cell viability using mass cytometry. Maxpar Metal-Conjugated Antibodies (Standard BioTools™) were used to identify all major T-cell subsets, perform comprehensive immunophenotyping of cytokine-expressing cells, and assess the activation status of these subtypes, as shown in **Supplementary Table 1**, which also includes the catalog numbers for each antibody. Data were obtained from the Helios mass cytometer (Fluidigm), and standard data were normalized. The results from each run were collected and analyzed using R and PhenoGraph, as previously described (6).

### Statistical analysis

The SPSS software package (version 23.0; SPSS Inc., USA) was used for all statistical analyses. Continuous variables are presented as the median (minimum – maximum), and categorical variables as the number of observations (n) and percentage (%). The Mann-Whitney U test, chi-square test, or Fisher’s exact test was applied to compare differences among groups. The significance level was set at P < 0.05 for two-sided tests. Spearman’s rank correlation was used for correlation analyses.

## Results

### Difference in neutralizing inhibition rate and anti-RBD levels in LTRs

**Figure 1:**
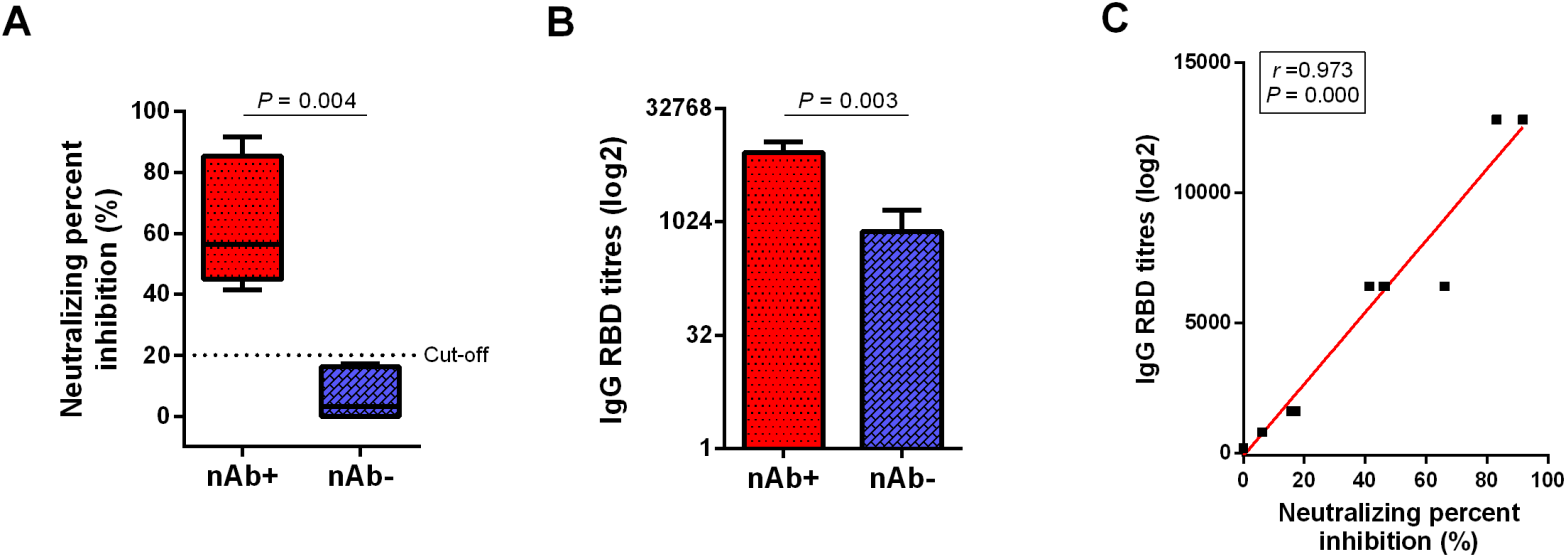
Comparison of antibody responses between the two groups of LTRs. **(A)** Neutralizing antibody detection in plasma from COVID-19 convalescent LTRs. The cutoff value is set at 20% signal inhibition. **(B)** Comparison of anti-RBD antibody responses between the nAb+ and nAb- groups. **(C)** Correlation between anti-RBD levels and NPI. p-values (two-sided) and r values are based on Spearman’s rank test. LTRs, liver transplant recipients; NPI, neutralizing percent inhibition.

Twelve LTRs who recovered from COVID-19 with mild symptoms were recruited. **Figure 1A** shows that LTRs were classified into two groups based on the neutralizing percent inhibition (NPI) results (P = 0.004): the group with Anti-SARS-CoV-2 nAb detected (nAb+, 6 patients, NIR ≥ 20%) and the group with nAb undetected (nAb-, 6 patients, NIR < 20%). Subsequent analysis was performed based on these two groups. Higher anti-RBD titers were detected in the nAb+ group compared to the nAb- group (P = 0.003, **Figure 1B**). NPI showed a strong correlation with anti-RBD levels (Spearman’s r = 0.973, P = 0.000, **Figure 1C**).

### T cell subsets in COVID-19 convalescent LTRs

**Figure 2:**
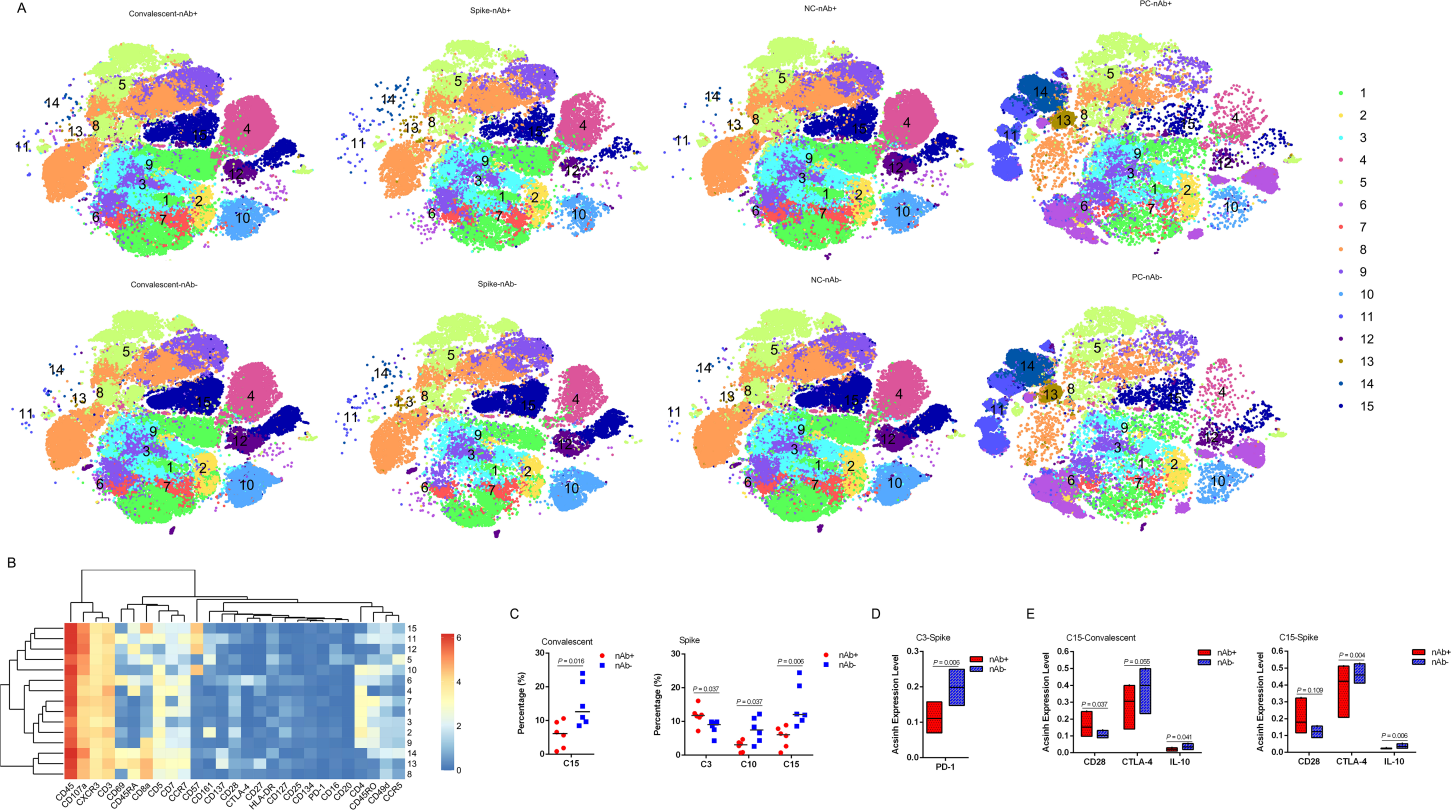
T cell subsets in LTRs recovered from COVID-19. **(A)** CyTOF-identified cell clusters from PBMCs visualized by t-distributed stochastic neighbor embedding (t-SNE). **(B)** Heatmap showing the expression patterns of various markers, stratified by FlowSOM clusters. The heat scale is calculated as the column z-score of mean fluorescence intensity (MFI). **(C)** Percentage of each cluster in the two groups. Each dot represents an individual group, with a line at the median of the groups. **(D)** The arcsinh expression level of PD-1 in the C3 subset after stimulation with the SARS-CoV-2 Spike Omicron XBB peptide pool, comparing nAb+ and nAb- LTRs. A line is drawn at the median of the groups. **(E)** The arcsinh expression levels of CD28, CTLA-4, and IL-10 in the C15 subset in convalescent and SARS-CoV-2-specific T cells. A line is drawn at the median of the groups. Significance was determined using the Kruskal–Wallis test. Statistical significance was set at a two-sided p-value <0.05. C3: Effector memory CD4+ - 1 (CD45RO+ CD28- CCR7-), C10: Effector memory CD4+ - 2 (CD57+ CD45RO+ CD28- CCR7-), C15: Terminal effector CD8+ - 3 (CD57+ CD8+ CD45RA+ CCR7-).

To investigate the cellular immune response to SARS-CoV-2, we first assessed T cell subsets according to nAb levels in peripheral blood derived from LTRs 6 months post-COVID-19 infection. Fifteen T cell populations were clustered (**Figures 2A, B**), including: C1_central memory CD4+ - 1 (CD4+ CD7+CD45RO+CCR7+), C2_transitional memory CD4+ (CD45RO+CD28+CCR7-), C3_effector memory CD4+ - 1 (CD45RO+CD28-CCR7-), C4_ Naïve CD4+ (CD4+CD45RA+CCR7+), C5_effector memory CD8+ (CD161+CD8+CD45RO+CCR7-), C6_stem cell memory CD4+ (CD69+CTLA-4+CD4+CD45RA+CCR7+CD28+), C7_central memory CD4+ - 2 (CD4+CD45RO+CCR7+), C8_Naive CD8+ (CD8+CD45RA+CCR7+), C9_DPT (CD4+mediumCD8+high), C10_effector memory CD4+ - 2 (CD57+CD45RO+CD28-CCR7-), C11_terminal effector CD8+ - 1 (CD69+CD8+CD45RA+CCR7-), C12_dNT (CD4-CD8-), C13_stem cell memory CD8+ (CD45RA+CCR7+CD28+), C14_terminal effector CD8+ - 2 (CD69+ CD137+CD8+CD45RA+CCR7-) and C15_terminal effector CD8+ - 3 (CD57+CD8+CD45RA+CCR7-). Nearly all T cell frequencies showed no significant differences between the two groups, except for C15_ terminal effector 3, which was decreased in the nAb+ group (P = 0.016) (**Figure 2C**). Interestingly, the nAb- convalescent LTRs showed a tendency for higher expression of CTLA-4 (P = 0.055), while the nAb+ group showed higher CD28 expression (P = 0.037). Additionally, elevated IL-10 expression was found in the nAb- group (P = 0.041) (**Figure 2E**).

### T cell response in LTRs recovered from COVID-19

To investigate how T cells mediate the memory response, we stimulated PBMCs from recovered LTRs with an XBB spike peptide pool from SARS-CoV-2 *in vitro*. SARS-CoV-2-specific T cell responses in peripheral blood from convalescent LTRs were compared according to nAb levels. We observed an elevation in C3_effector memory CD4 + 1 cells in the nAb+ group, which had lower PD-1 expression (P = 0.006) compared to the nAb- group (**Figures 2A–D**). We also detected a reduction in C10_effector memory CD4+ - 2 cells and C15_terminal effector CD8+ - 3 cells in the nAb+ group (**Figure 2C**). During the SARS-CoV-2-related T cell memory response, the C15_terminal effector CD8+ - 3 subset showed high expression of CTLA-4 (P = 0.004) and IL-10 (P = 0.006) but not CD28 (P = 0.109), similar to the corresponding subset in the nAb- group (**Figure 2E**).

### SARS-CoV-2- reactive T cell response in LTRs recovered from COVID-19

**Figure 3:**
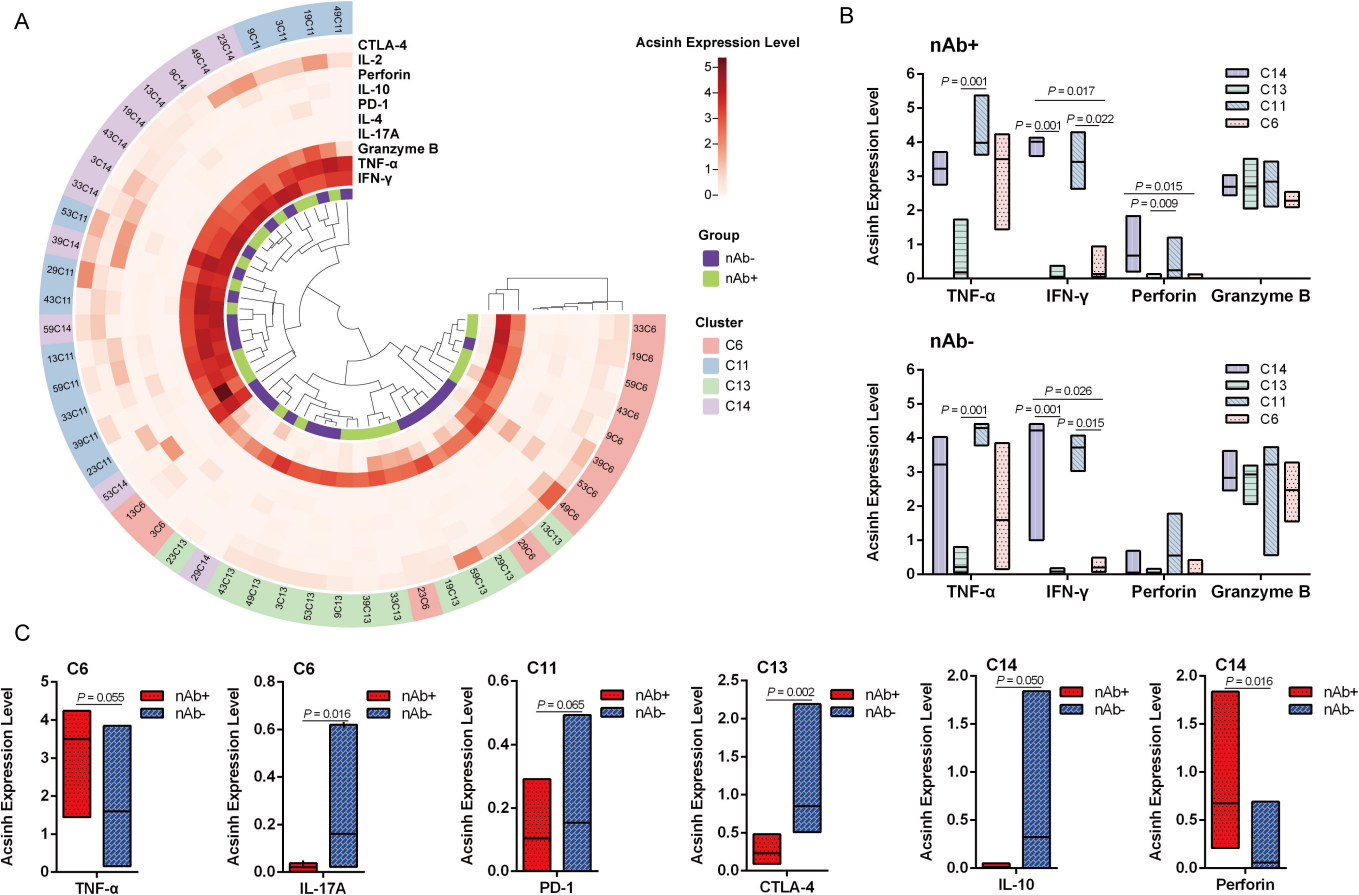
SARS-CoV-2-specific T cell response in convalescent LTRs. **(A)** Heatmap of arcsinh expression of IL-2, IL-4, IL-10, IL-17A, TNF-α, IFN-γ, perforin, granzyme B, PD-1, and CTLA-4 in four types of SARS-CoV-2-specific T cells. **(B)** The arcsinh expression levels of TNF-α, IFN-γ, perforin, and granzyme B in the four SARS-CoV-2-specific T cell clusters. A line is drawn at the median of the groups. **(C)** The arcsinh expression levels of TNF-α and IL-17A in C6, PD-1 in C11, CTLA-4 in C13, and IL-10 and perforin in C14. Significance was determined using the Kruskal–Wallis test, followed by multiple pairwise comparisons. Statistical significance was set at a two-sided p-value <0.05 and adjusted p < 0.05. C6: Stem cell memory CD4+ (CD69+ CTLA-4+ CD4+ CD45RA+ CCR7+ CD28+), C11: Terminal effector CD8+ - 1 (CD69+ CD8+ CD45RA+ CCR7-), C13: Stem cell memory CD8+ (CD45RA+ CCR7+ CD28+), C14: Terminal effector CD8+ - 2 (CD69+ CD137+ CD8+ CD45RA+ CCR7-).

SARS-CoV-2-reactive T cells in LTRs were considered as CD3+ CD4+ or CD3+ CD8+ T lymphocytes expressing CD137 and CD69 simultaneously (11). We identified four SARS-CoV-2-reactive T cell subsets in liver transplant recipients recovered from COVID-19, classified as C6, C11, C13, and C14 (**Figure 2B**). Next, the detection of all intracellular cytokines, including IL-2, IL-4, IL-10, IL-17A, TNF-α, IFN-γ, perforin, granzyme B, and negative regulators such as PD-1 and CTLA-4, was focused on these four SARS-CoV-2-reactive T cell subsets (**Figure 3A**). TNF-α and IFN-γ were highly expressed in C11_terminal effector CD8+ - 1, and IFN-γ was also enriched in C14_terminal effector CD8+ - 2 in both groups (**Figure 3B**, P < 0.05). Perforin was mainly detected in C11_terminal effector CD8+ - 1 and C14_terminal effector CD8+ - 2 in the nAb+ group (**Figure 3B**, P < 0.05). Granzyme B was expressed in all four types of SARS-CoV-2-specific T cells (**Figure 3A**). In the nAb- group, higher expression levels of IL-17A in C6_stem cell memory CD4+ (P = 0.016) and CTLA-4 in C13_stem cell memory CD8+ (P = 0.002) were observed, along with trends of increased PD-1 expression in C11_terminal effector CD8+ - 1 (P = 0.065) and IL-10 expression in C14_terminal effector CD8+ - 2 (P = 0.050) (**Figure 3C**). In the nAb+ group, elevated expression of perforin in C14_terminal effector CD8+ - 2 (P = 0.016) and a trend toward increased TNF-α expression in C6_stem cell memory CD4+ (P = 0.055) were also observed (**Figure 3C**).

## Discussion

The generation and persistence of memory T cells specific to SARS-CoV-2 are crucial for long-term immunity and play a central role in understanding reinfection cases and the longevity of vaccine-mediated protection. Therefore, it is essential to assess the ability of adaptive immune memory in liver transplant recipients (LTRs) who have recovered from COVID-19. In this study, convalescent LTRs were classified into two groups based on the detection of neutralizing antibodies (nAb) to evaluate the memory T cell response to SARS-CoV-2 in LTRs who were 6 months post-infection.

As demonstrated, SARS-CoV-2-specific T cell immunity is elicited in most LTRs but declines over time, and it is comparable to that observed in immunocompetent patients (12). Our results showed that the nAb+ group exhibited higher anti-RBD antibody titers than the nAb- group, with a strong association between NPI and anti-RBD levels in convalescent LTRs. Previous reports have also shown a strong correlation between the levels of antibodies that bind to the RBD of SARS-CoV-2 and the neutralizing antibodies in patients with SARS-CoV-2 infection (13, 14).

The difference in cellular immunity between robust and poor immune responses after SARS-CoV-2 infection is a major concern in organ transplant patients. Therefore, it is necessary to reveal the memory response mediated by T cells in LTRs. We performed high-parameter CyTOF analysis to examine the peripheral T cell immune spectrum before and after spike stimulation of PBMCs from recovering LTRs and to assess the phenotypes and intracellular cytokine levels. During convalescence from COVID-19 in LTRs, the frequencies of T cell subsets were similar between the nAb+ and nAb- groups, except for C15_terminal effector CD8+ - 3, which was lower in the nAb+ group. CD8+CD57+ T cells, a subset with strong cytotoxic potency and impaired proliferative capability (15), may be activated and depleted in the early stages of SARS-CoV-2 infection, leading to their decline in the nAb+ group. As the virus is cleared and nAb is produced, the demand for and activation of CD57+CD8+ terminal effectors diminished.

A similar T cell response was exhibited by LTRs and non-transplanted individuals one year after COVID-19 diagnosis (11). The majority of individuals who had recovered from SARS-CoV-2 infection 6 months ago showed SARS-CoV-2-specific T cell responses (16). We first obtained the complete T cell immune response landscape for LTRs after stimulation with the Spike peptide pool. Some differences were observed between the nAb+ and nAb- groups. We found an elevation in C3_effector memory CD4+ - 1 cells in the nAb+ group, with lower PD-1 expression. This suggests that the nAb+ group had more CD4+ effector memory cells, stimulated by the spike peptide pool, entering a memory state and exhibiting stronger antigen-specific immune responses. PD-1, an immune checkpoint molecule, interacts with PD-L1 on T cells, attenuating T cell activation and proliferation, and influencing T cell differentiation and function, leading to the formation of memory T cells or regulatory T cells (17). It has been reported that antigen stimulation can induce CD4+ T cell exhaustion, which is characterized by a reduced number of cells, impaired cytokine secretion, and increased expression of inhibitory receptors such as PD-1, Lag3, and CTLA-4 (18). Additionally, we observed a reduction in C10_effector memory CD4+ - 2 cells and C15_terminal effector CD8+ - 3 cells in the nAb+ group. CD57 expression has been reported to mark replicative senescence and antigen-triggered apoptotic death of CD8+ T cells (19).

In the nAb- group, we also found high expression of CTLA-4 and IL-10 in the C15_terminal effector CD8+ - 3 subset, similar to the corresponding subset before spike stimulation. These findings suggest that antigen stimulation and immune memory affect the differentiation and function of T cells in recovered LTRs. The contrasting CTLA-4 and CD28 expression profiles between the nAb- and nAb+ groups may reflect differences in immune activation and regulation following SARS-CoV-2 exposure in liver transplant recipients. The nAb- individuals, with higher CTLA-4 expression, may have a more regulated or exhausted immune response, limiting the effectiveness of their T cell and antibody-mediated immunity against SARS-CoV-2. In contrast, nAb+ individuals, with elevated CD28 expression, may represent a subset with more robust immune activation, capable of both neutralizing antibody production and T cell-mediated immunity.

The T cell response in nAb- patients to SARS-CoV-2 antigen stimulation was different from that in the nAb+ group. The T cells in the nAb- group had not established effective immune memory and could not respond quickly to SARS-CoV-2 antigen stimulation. They required more co-stimulatory signals to activate and expand, a process regulated by IL-10, a cytokine that can both inhibit and promote T cell effector function and exhaustion (20).

CD8+ T cells play a crucial role in controlling and resolving viral infections, with their phenotypes and effector functions varying depending on the inflammatory context and the duration and extent of antigen exposure (21). In line with previous studies, SARS-CoV-2-reactive T cells in LTRs were considered as CD3+CD4+ or CD3+CD8+ T lymphocytes expressing both CD137 and CD69 simultaneously (11). We found that the SARS-CoV-2-reactive T cells were divided into four clusters. TNF-α and IFN-γ were highly expressed in C11_terminal effector CD8+ - 1 and C14_terminal effector CD8+ - 2 in both groups, indicating that these two clusters played an important role in the early stages of infection but may gradually decrease or disappear afterward. Perforin was mainly detected in C14_terminal effector CD8+ - 2 in the nAb+ group, suggesting that this subset exhibited stronger cytotoxicity and could effectively eliminate virus-infected cells, providing immune protection (22). Different intracellular cytokines and negative regulators’ expression patterns in the four SARS-CoV-2-reactive T cells between the nAb+ and nAb- groups suggest that T cells in the nAb+ group have stronger killing and memory capabilities and can effectively clear the virus, while T cells in the nAb- group exhibit stronger inflammation and suppression, possibly due to excessive activation and inflammatory responses of T cells.

This study has several limitations that should be considered. First, the relatively small number of LTRs may have limited the robustness of the conclusions. Second, we did not recruit healthy volunteers with similar infectious conditions for this study. In a separate study, LTRs and healthy donors with positive nAb showed a similar immune response after receiving two doses of vaccination (6). Future studies will examine different infectious patterns, such as infection only, vaccination after infection, and infection after different vaccination strategies, to better understand immune memory in LTRs. Despite these limitations, our study demonstrates that convalescent LTRs with different immune memory patterns induced by SARS-CoV-2 exhibit distinct T cell responses, particularly those with negative nAb, who showed milder cellular responses and stronger exhaustion status. This highlights the need to pay more attention to the immune status of nAb- convalescent LTRs facing new variants of SARS-CoV-2 and to implement effective protective strategies in a timely manner.

## Data Availability

The original contributions presented in the study are included in the article/**Supplementary Material**. Further inquiries can be directed to the corresponding authors.

## References

[1] Dumortier J Duvoux C Roux O Altieri M Barraud H Besch C. Covid-19 in liver transplant recipients: the French SOT COVID registry. Clin Res Hepatol Gastroenterol. (2021) 45:101639. doi: 10.1016/j.clinre.2021.101639 33636654 PMC7843027

[2] Rabinowich L Grupper A Baruch R Ben-Yehoyada M Halperin T Turner D. Low immunogenicity to SARS-CoV-2 vaccination among liver transplant recipients. J Hepatol. (2021) 75:435–8. doi: 10.1016/j.jhep.2021.04.020 PMC8058047 33892006

[3] Holden IK Bistrup C Nilsson AC Hansen JF Abazi R Davidsen JR. Immunogenicity of SARS-CoV-2 mRNA vaccine in solid organ transplant recipients. J Intern Med. (2021) 290:1264–7. doi: 10.1111/joim.v290.6 PMC8447120 34237179

[4] Harberts A Schaub GM Ruether DF Duengelhoef PM Brehm TT Karsten H. Humoral and cellular immune response after third and fourth SARS-coV-2 mRNA vaccination in liver transplant recipients. Clin Gastroenterol Hepatol. (2022) 20:2558–66. doi: 10.1016/j.cgh.2022.06.028 PMC9287575 35850415

[5] Luxenburger H Reeg DB Lang-Meli J Reinscheid M Eisner M Bettinger D. Boosting compromised SARS-CoV-2-specific immunity with mRNA vaccination in liver transplant recipients. J Hepatol. (2023) 78:1017–27. doi: 10.1016/j.jhep.2023.02.007 PMC10019593 36804404

[6] Duan B Zhang G Wang W Yin J Liu M Zhang J. Immunogenicity profiling and distinct immune response in liver transplant recipients vaccinated with SARS-CoV-2 inactivated vaccines. Front Immunol. (2022) 13:954177. doi: 10.3389/fimmu.2022.954177 36189318 PMC9517166

[7] Caballero-Marcos A Salcedo M Alonso-Fernandez R Rodriguez-Peralvarez M Olmedo M Graus MJ. Changes in humoral immune response after SARS-CoV-2 infection in liver transplant recipients compared to immunocompetent patients. Am J Transplant. (2021) 21:2876–84. doi: 10.1111/ajt.16599 PMC8251470 33835707

[8] Noh JY Jeong HW Kim JH Shin EC. T cell-oriented strategies for controlling the COVID-19 pandemic. Nat Rev Immunol. (2021) 21:687–8. doi: 10.1038/s41577-021-00625-9 PMC8424399 34497383

[9] Tu ZH Jin PB Chen DY Chen ZY Li ZW Wu J. Evaluating the response and safety of inactivated COVID-19 vaccines in liver transplant recipients. Infect Drug Resist. (2022) 15:2469–74. doi: 10.2147/IDR.S359919 PMC9112169 35592105

[10] Kulkarni AV Jaggaiahgari S Iyengar S Simhadri V Gujjarlapudi D Rugwani H. Poor immune response to coronavirus disease vaccines in decompensated cirrhosis patients and liver transplant recipients. VACCINE. (2022) 40:6971–8. doi: 10.1016/j.vaccine.2022.10.042 PMC9595300 36374707

[11] Citores MJ Caballero-Marcos A Cuervas-Mons V Alonso-Fernandez R Graus-Morales J Arias-Milla A. Long term SARS-CoV-2-specific cellular immunity after COVID-19 in liver transplant recipients. J Microbiol Immunol Infect. (2023) 56:526–36. doi: 10.1016/j.jmii.2023.03.003 PMC10020132 36964052

[12] Fernandez-Ruiz M Olea B Almendro-Vazquez P Gimenez E Marcacuzco A San JR. T cell-mediated response to SARS-CoV-2 in liver transplant recipients with prior COVID-19. Am J Transplant. (2021) 21:2785–94. doi: 10.1111/ajt.16708 PMC8222887 34092033

[13] Premkumar L Segovia-Chumbez B Jadi R Martinez DR Raut R Markmann A. The receptor binding domain of the viral spike protein is an immunodominant and highly specific target of antibodies in SARS-CoV-2 patients. Sci Immunol. (2020) 5(48):eabc8413. doi: 10.1126/sciimmunol.abc8413 32527802 PMC7292505

[14] Ni L Ye F Cheng ML Feng Y Deng YQ Zhao H. Detection of SARS-coV-2-specific humoral and cellular immunity in COVID-19 convalescent individuals. IMMUNITY. (2020) 52:971–7. doi: 10.1016/j.immuni.2020.04.023 PMC7196424 32413330

[15] Huang B Liu R Wang P Yuan Z Yang J Xiong H. CD8(+)CD57(+) T cells exhibit distinct features in human non-small cell lung cancer. J Immunother Cancer. (2020) 8(1):e000639. doi: 10.1136/jitc-2020-000639 32606053 PMC7328901

[16] Long QX Jia YJ Wang X Deng HJ Cao XX Yuan J. Immune memory in convalescent patients with asymptomatic or mild COVID-19. Cell Discovery. (2021) 7:18. doi: 10.1038/s41421-021-00250-9 33767156 PMC7993859

[17] Jubel JM Barbati ZR Burger C Wirtz DC Schildberg FA. The role of PD-1 in acute and chronic infection. Front Immunol. (2020) 11:487. doi: 10.3389/fimmu.2020.00487 32265932 PMC7105608

[18] Kunzli M Masopust D. CD4(+) T cell memory. Nat Immunol. (2023) 24:903–14. doi: 10.1038/s41590-023-01510-4 PMC10343737 37156885

[19] Brenchley JM Karandikar NJ Betts MR Ambrozak DR Hill BJ Crotty LE. Expression of CD57 defines replicative senescence and antigen-induced apoptotic death of CD8+ T cells. BLOOD. (2003) 101:2711–20. doi: 10.1182/blood-2002-07-2103 12433688

[20] Couper KN Blount DG Riley EM. IL-10: the master regulator of immunity to infection. J Immunol. (2008) 180:5771–7. doi: 10.4049/jimmunol.180.9.5771 18424693

[21] Kuhn R Sandu I Agrafiotis A Hong KL Shlesinger D Neimeier D. Clonally expanded virus-specific CD8 T cells acquire diverse transcriptional phenotypes during acute, chronic, and latent infections. Front Immunol. (2022) 13:782441. doi: 10.3389/fimmu.2022.782441 35185882 PMC8847396

[22] Makedonas G Hutnick N Haney D Amick AC Gardner J Cosma G. Perforin and IL-2 upregulation define qualitative differences among highly functional virus-specific human CD8 T cells. PloS Pathog. (2010) 6:e1000798. doi: 10.1371/journal.ppat.1000798 20221423 PMC2832688

