# Supplementary material for: Distinct immune memory induced by SARS-CoV-2 in convalescent liver transplant recipients
Source: Front Immunol. 2025 Apr 2;16:1420150. doi: 10.3389/fimmu.2025.1420150 (PMC12000081; doi:10.3389/fimmu.2025.1420150)

**Supplementary data**

**Supplementary Table 1 Mass cytometry Panel Design**

| Marker | Channel | Staining | Standard BioTools™ PN |
| --- | --- | --- | --- |
| CD69 | 113Cd | Surface | 3113002C |
| CD107a | 106Cd | Surface | 3106002C |
| CTLA-4 | 162Dy | Intracellular | 3162039C |
| Granzyme B | 198Pt | Intracellular | 3198002C |
| IFNγ | 116Cd | Intracellular | 3116002C |
| IL-2 | 112Cd | Intracellular | 3112002C |
| Perforin | 196Pt | Intracellular | 3196002C |
| TNFα | 114Cd | Intracellula | 3114002C |
| CD4 | 145Nd | Surface | 3145001B |
| CD8a | 146Nd | Surface | 3146003B |
| CD16 | 148Nd | Surface | 3148004B |
| CD25 | 149Sm | Surface | 3149010B |
| CD45 | 154Sm | Surface | 3154001B |
| CD45RO | 165Ho | Surface | 3165011B |
| CD27 | 167Er | Surface | 3167002B |
| CD45RA | 169Tm | Surface | 3169008B |
| CD3 | 170Er | Surface | 3170001B |
| CD57 | 172Yb | Surface | 3172009B |
| HLA-DR | 174Yb | Surface | 3174001B |
| CD127 | 176Yb | Surface | 3176004B |
| CD5 | 143Nd | Surface | 3143007B |
| CD7 | 147Sm | Surface | 3147006C |
| CD28 | 160Gd | Surface | 3160003B |
| CD49d | 141Pr | Surface | 3141004C |
| CD161 | 164Dy | Surface | 3164009C |
| CCR7 | 159Gd | Surface | 3159003C |
| CCR5 | 144Nd | Surface | 3144007C |
| PD-1 | 155Gd | Intracellular | 3155009C |
| IL-4 | 163Dy | Intracellular | 3163011C |
| IL-10 | 166Er | Intracellular | 3166008C |
| IL-17A | 161Dy | Intracellular | 3161008C |
| CD137/4-1BB | 209Bi | Intracellular | 3209015B |
| CD20 | 171Yb | Surface | 3171012C |
| CD134/OX40 | 150Nd | Intracellular | 3150023C |

**Suggested gating strategy for T cell subsets**


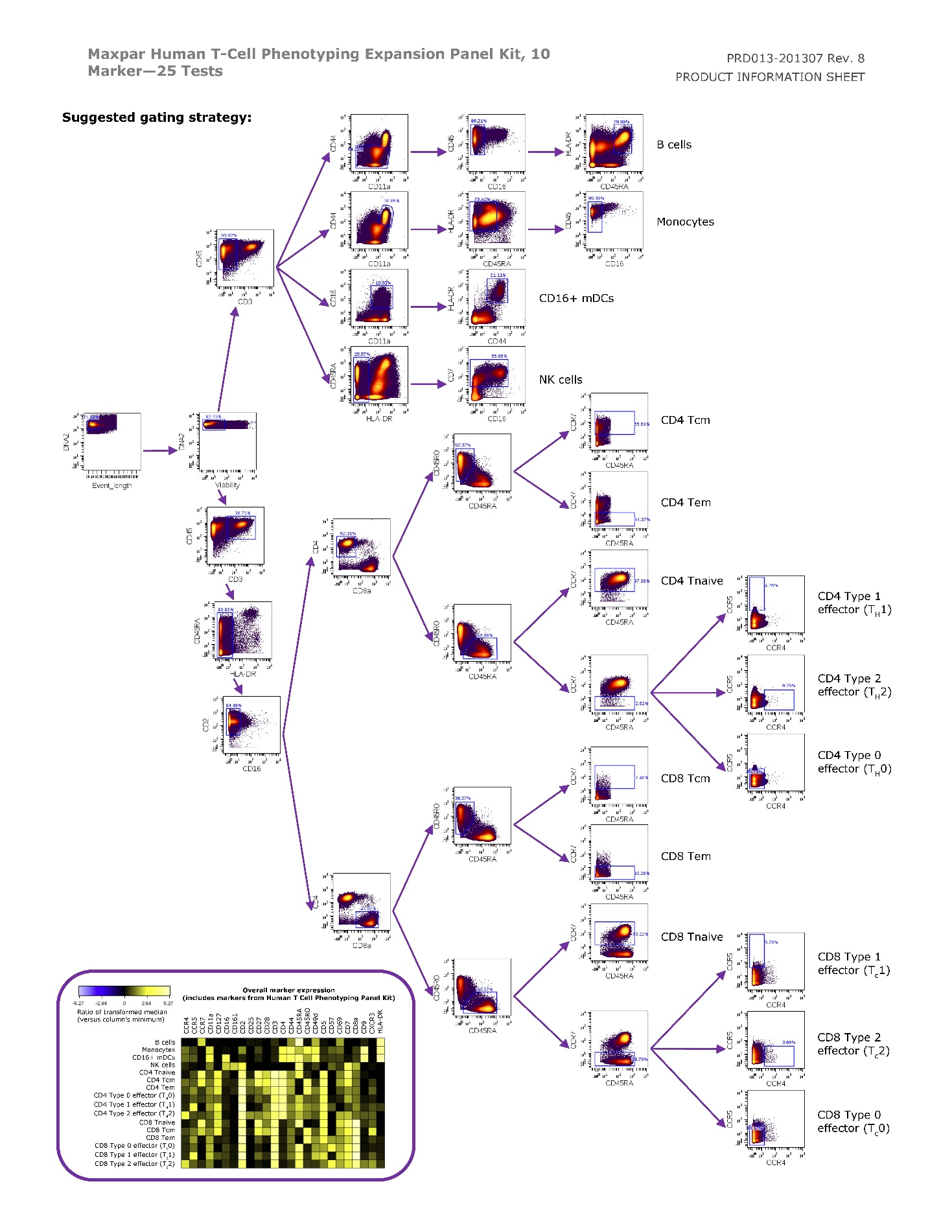

Supplement: Supplementary file 1 [file DataSheet1.docx]
